# Group cognitive behavioural therapy can reduce stigma and improve treatment compliance in major depressive disorder patients

**DOI:** 10.1111/eip.12841

**Published:** 2019-07-02

**Authors:** Ping Tong, Ping Bu, Yang Yang, Liping Dong, Ting Sun, Yuanhong Shi

**Affiliations:** ^1^ Department of Clinical Psychology Clinical Medical College of Yangzhou University Yangzhou People's Republic of China; ^2^ Department of Integrated Traditional Chinese and Western Medicine Medical College of Yangzhou University Yangzhou People's Republic of China

**Keywords:** cognitive behavioural therapy, group therapy, major depressive disorder, stigma, treatment compliance

## Abstract

**Aim:**

The stigma of mental disorders and poor treatment compliance can deprive patients with major depressive disorder (MDD) of receiving standard treatment. This study aimed to clarify whether MDD patients' stigma and treatment non‐compliance issues can be mitigated using group cognitive behavioural therapy (GCBT).

**Methods:**

Eighty‐eight participants with first‐episode MDD were randomly divided into GCBT groups (GCBTs) and control groups (Cs). The Hamilton Rating Scale for Depression (HRSD‐24), Morisky Medication Adherence Scale (MMAS‐8™) and Stigma Scale (SS) were used to evaluate the therapeutic effect on all participants before and after receiving GCBT. Data were assessed at baseline and post‐treatment.

**Results:**

At the baseline, there were no significant differences (in terms of the demographic data of the participants and the scores on HRSD‐24, MMAS‐8™ and SS) between the two groups. After 8 weeks of GCBT, there were significant differences in HRSD‐24 (*P* < .01), MMAS‐8™ (*P* < .01), SS (*P* < .01), treatment compliance (*P* < .01) and therapeutic effect evaluation based on rate of deduction (*P* < .05) between the two groups.

**Conclusion:**

GCBT can reduce patients' sense of stigma, improve treatment compliance, effectively alleviate depressive symptoms and promote the recovery of MDD patients.

## 1 INTRODUCTION

The word “stigma” originated from the Greek word “stizein” and has the meaning of tattoo. The concept of “stigma” was originally mentioned in sociology by Erving Goffman as “an attribute that is deeply discrediting, and the stigmatized individual is reduced from a whole and usual person to a tainted or discounted one.” Thus, it mainly refers to the significantly different physiological and behavioural characteristics that patients exhibit; these behaviours can attract negative or wrong social attitudes towards the patients, and thus, the patients often lose their social value and reputation in the eyes of others (Goffman, 1963).

Stigma includes two aspects: public stigma and self‐stigma. Public stigma refers to the negative perception and the discrimination and/or isolation the patient suffers on account of others; that is, people with mental disorders are thought to be unpopular in society, and the negative perception of mental disorders can influence family and community behaviours towards such patients (Corrigan, Watson, & Miller, 2006). Sometimes even medical staff members exhibit public stigma. Such social attitudes, prejudices and actions can cause family members and the patients themselves to feel ashamed.

Self‐stigma refers to the internalized shame of a particular group; this shame is often caused by negative stereotypes of oneself or the feelings of denial that occur when one is being stigmatized by others. The stigmatized person may internalize the cognitive bias, and thus develop a negative feeling towards himself/herself. Self‐stigma develops as a result of a decrease in self‐esteem and an increase in depression. Patients are often ashamed and embarrassed by the symptoms of their depression (Abiri, Oakley, Hitchcock, & Hall, 2016; Corrigan, 2004).

Patients with Major Depressive Disorders (MDDs) often experience strong feelings of self‐stigma about their disorder. In addition, the cognitive impairment caused by MDD often strengthens their sense of stigma. Such stigma can have a greater impact on the outcome of MDD. On the one hand, patients with strong stigma attempt to hide their disease and avoid contact with others. When the label “You need help” is used, these patients may descend into further low self‐esteem and develop an impaired sense of self‐esteem. They may form distorted cognitions and develop maladaptive coping strategies, internalizing thoughts such as “Only the weak will seek help” and “Man should not give in to any discomfort”. Thus, they avoid treatment, and consequently, their MDD symptoms become aggravated. Patients tend to attribute the cause of the disease to their own personality defects or incapability (Midgley et al., 2017; Picco et al., 2017).

Treatment compliance refers to the compliance (or non‐compliance) of patients with their doctors' instructions and the degree to which the patients' treatment behaviour is consistent with such instructions. Research (Tang & Wen, 2015) has found that, the treatment compliance of patients with MDD is closely related to their attitude towards the disorders; that is, they tend to have poor treatment compliance if they have a stigma about their disorder. Treatment compliance and stigma not only affect the outcome of treatment but also lead to negative emotional experiences for the patients, causing them to cover up their inappropriate behaviour, and thus aggravate their depressive symptoms. Studies have also found that, as peoples' knowledge about MDD increases, their stigmatizing attitude towards it decreases (Townsend et al., 2017).

**Figure 1:**
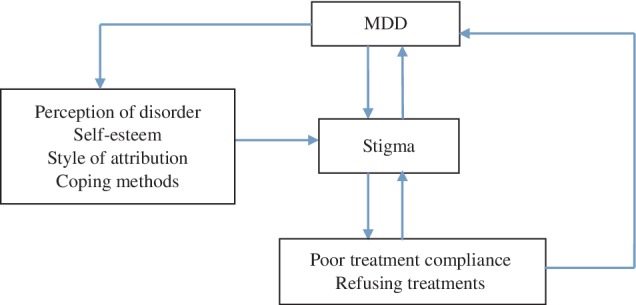
Interaction between stigma and treatment compliance in major depressive disorder (MDD) patients

We can draw a conclusion, from the abovementioned facts, that there is a negative correlation between stigma and treatment compliance. Patients with higher levels of stigma‐related negative feelings will have a higher tendency to avoid receiving treatment. When attitudes of acceptance towards MDD are high, it becomes easier for MDD sufferers to receive medication and psychotherapy (Carrara & Ventura, 2018; Latalova, Kamaradova, & Prasko, 2014; Tucker et al., 2013), especially in the early phase of the disorder (Firmin et al., 2018; Kular et al., 2018); that is, the feelings of stigma affect the outcome of the disorder by affecting the treatment compliance of MDD patients. The possible mechanism underlying this tendency is shown in Figure 1. Thus, finding ways to reduce the patient's stigma and improve treatment compliance are very important issues for the MDD treatment process.

Group Cognitive Behavioural Therapy (GCBT) is widely used in clinical practice because of its high cost‐time benefits and potential positive effects (Okumura & Ichikura, 2014). Several studies have shown that people with mental disorders such as schizophrenia, MDD, general anxiety disorder, obsessive‐compulsive disorder and even comorbidity of physical disease can benefit from GCBT (Archer et al., 2016; Butler et al., 2018; Strauss, Huppert, Simpson, & Foa, 2018). This study is the first to use GCBT, focusing on the stigma and treatment compliance of MDD patients, and exploring the therapeutic effect of GCBT on the outcomes of the disease treatment process. The following sections outline the research process.

## 2 MATERIALS AND METHODS

### 2.1 Participants

Between January and August 2018, 90 patients with the first episode of MDD were recruited voluntarily as participants from the Clinical Medical College of Yangzhou University's psychology clinic. The subjects were randomly assigned to the GCBT group (GCBT) or the control group (Cs) by using a random number table. The study was approved by the ethics committee of the Clinical Medical College of Yangzhou University and conducted in accordance with the Declaration of Helsinki. All participants were randomly divided into two groups.

The final participant group included participants who (a) were all at least 18 years old but not more than 60 years old (gender was not a limit), (b) had an educational level of at least junior high school, (c) scored above 20 points on the 24 items of the Hamilton Rating Scale for Depression (HRSD‐24) and (d) met the diagnostic criteria for major depressive episodes according to The Diagnostic and Statistical Manual of Mental Disorders, Fifth Edition (American Psychiatric Association, 2013).

Potential participants were excluded if they (a) presented severe physical diseases or their symptoms, (b) had depressive symptoms caused by physical disease or other mental disorders, (c) had a history of substance abuse and (d) had a history of other mental disorders.

### 2.2 Evaluations

#### 2.2.1 Hamilton Rating Scale for Depression

All the participants were subjected to HRSD‐24 (Zhang, 1998) evaluation before and after GCBT. HRSD‐24 contains 24 variables, which are measured on a 5‐point scale and are used to assess depression severity. Participants with a score equal to or above 20 were considered to have severe depressive symptoms of an ongoing episode. Compiled by Hamilton in 1967 (Hamilton, 1967), it is the most commonly used scale for assessing depression, with a reliability coefficient (r) of 0.99. The reliability for the symptom score was 0.78 to 0.98. HRSD‐24 can be used to evaluate therapeutic effect based on the rate of deduction (RD) of the scores of HRSD‐24. If the RD is 75% or above, it indicates that the therapeutic effect has healing value, 50% to 75% indicates a more progressive effect, 25% to 50% indicates a less progressive effect, and less than 25% is considered invalid.

Physicians and researchers who had been trained to perform psychological evaluations carried out the test. Before and after GCBT, the participants were assessed for depressive symptoms and treatment effects based on the HRSD‐24.

#### 2.2.2 Morisky Medication Adherence Scale ™(© 2006 Donald E. Morisky)*

The Morisky Medication Adherence Scale (MMAS‐8™) was created by Morisky, Ang, Krousel‐Wood, and Ward (2008), and was introduced to China by Yang et al. (2014) (Bahar et al., 2014). Yang et al revised the version for the Chinese context, being careful to maintain good reliability and validity. This scale consists of eight items. The alternative answers for items 1 to 7 are “yes” and “no”. The “yes” and “no” of each question is given a different score. The fifth item is rated in a reversely order and the eighth item's alternative answers are “never”, “occasionally”, “sometimes”, “frequently” and “all the time”, which is given a different score, respectively. There are three ranks of the MMAS‐8™ according to the scores: poor compliance, moderate compliance, and good compliance.

#### 2.2.3 Stigma Scale

Zeng, He, and Tian (2009) adapted the Stigma Scale for use with Chinese MDD patients. The scale has 32 items, and 0 to 3 points are used to rank the different degrees of stigma. The higher the score, the higher is the stigma. The items of the scale are divided into social factors, ability factors and treatment factors. The Cronbach coefficient for the scale is 0.9, and the internal consistency coefficient of the three factors is between 0.68 and 0.85. Therefore, scale has good reliability and validity.

### 2.3 Intervention

All the participants were divided into two groups: GCBT group patients (GCBTs; *n* = 45) and control group patients (Cs; *n* = 45). In the treatment process of the GCBTs, two patients dropped out: one participant experienced suicidal behaviour due to aggravation of the condition and was therefore hospitalized and another participant was dissatisfied with the treatment and requested to be transferred to another hospital for treatment. The remaining 43 individuals completed the treatment. There were five subgroups in each group: eight cases in two subgroups for GCBTs and nine cases in the other subgroups including the Cs.

In addition to administration of the same antidepressant, the GCBTs received GCBT once a week for 60 minutes for an 8‐week period (Beck, 2011; Wang, Yuan, & Sun, 2015), while the Cs waited for GCBT. After follow‐up, the Cs were offered the same GCBT as the GCBTs at our clinic. The content of the GCBT was focused on helping patients acquire knowledge about their MDD, teaching them methods to identify and modify irrational thoughts that might lead to low self‐esteem and stigma, helping them enhance their problem‐solving strategies and decision‐making skills so that they would be able to deal with the practical issues and aspects of MDD, and managing medication use and favourable behaviours. The details of GCBT are shown in Table 1. GCBT quality control was carried out as follows: Four psychotherapists conducted the GCBT by dividing into two groups and randomizing the GCBTs for conducting GCBT. The whole process was monitored by a supervisor, and all the GCBT providers received weekly supervision.

**Table 1:** Outline of GCBT

Session	Main subject	Content
1	Therapeutic alliance	Build therapeutic alliance Introduce GCBT program and therapeutic principles
2	Knowledge on MDD	Discuss the symptoms and pathogenesis of MDD Effects of emotion, thoughts and behaviours on MDD
3	Stigma and MDD	Discuss the definition of stigma, causes and harms and strategies to eliminate stigma
4	Treatment compliance	Focus on importance of treatment compliance Strategies to enhance treatment compliance
5	Medicine management and self‐management	Discuss importance of rational use of drugs and self‐management
6	Emotion, thoughts and behaviours	The role of emotion, behaviours and thoughts Identify dysfunctional thoughts, emotion and behaviours of MDD
7	Rebuilding cognition	Train patients cognitive rebuilding skills and techniques for modifying irrational thoughts that may affect their emotions and deduced or deteriorated MDD
8	Problem solving and discuss the future	Explain the strategy of solving the problem Discuss the future Strengthen consolidation, help patients rebuild confidence Farewell

Abbreviations: GCBT, group cognitive behavioural therapy; MDD, major depressive disorder.

The Cs received the same eight GCBT treatment sessions as the GCBTs after the research. The following rule was enforced strictly: If any participants experienced any discomfort that caused the inability to continue GCBT treatment, he/she could withdraw from the study at any time. Assessments during the intervention were performed before and after the GCBT, and a rater‐blind approach was used; the professional staff members, who did not know any of the study participants, used group‐testing to complete the baseline of the GCBTs and Cs, respectively.

### 2.4 Statistical analysis

Statistical Product and Service Solutions 17.0 was employed to complete the analyses. Age, HRSD‐24, MMAS‐8™ and SS were analysed using independent‐samples *t* test. Gender, education, marriage and family history were compared using the *χ* ^2^ test. Paired‐samples *t* test was used to compare the scale scores before and after GCBT. Rank sum test was used to compare the different levels of treatment compliance and therapeutic effects. *P* < .05 was considered to indicate statistical significance.

## 3 RESULTS

### 3.1 Demographic data of participants

Table 2 shows the detailed demographic information of all the participants. Results of statistical analysis of age, gender, education, marriage and family history showed no significant differences between GCBTs and Cs (*P* > .05).

**Table 2:** The demographic data of the participants (*M* ± *S*/*n* [%])

		GCBTs (*n* = 43)	Cs (*n* = 45)	*χ*2(*t*)‐value	*P*‐value
Age(years)		38.67 ± 13.17	36.82 ± 83	0.62^a^	.54
Gender(*n*/[%])	Male	12 (27.90)	13 (28.89)	0.01	.92
	Female	31 (72.09)	32 (71.11)		
Education (*n*/[%])	High school and below	14 (32.56)	17 (37.78)	0.45	.80
	Bachelor	26 (60.47)	26 (57.78)		
	Master and above	3 (6.98)	2 (4.44)		
Marriage (*n*/[%])	Married	30 (69.77)	30 (66.67)	0.10	.76
	Single	13 (30.23)	15 (33.33)		
Family history (*n*/[%])	With	8 (18.60)	9 (20.00)	0.03	.87

a Is the *t* value.

### 3.2 Comparison of baseline scores and ranks between GCBTs and Cs

Table 3 shows that, at the baseline, there were no significant differences in terms of HRSD‐24, MMAS‐8™, SS, factor scores for SS and ranks of compliance between the GCBTs and Cs (*P* > .05).

**Table 3:** Comparison of baseline scores and ranks between GCBTs and Cs(*M* ± *S*/*n* [%])

		GCBTs (*n* = 43)	Cs (*n* = 45)	*t*(Z)‐value	*P*‐value
HRSD		30.16 ± 5.10	29.73 ± 5.10	0.40	.69
MMAS‐8		4.87 ± 1.55	4.84 ± 1.39	0.07	.95
Ranks	Good compliance	0 (0.00)	0 (0.00)		
	Moderate compliance	9 (20.93)	9 (20.00)	0.11^a^	.91
	Poor compliance	34 (79.07)	36(80)		
SS	Social factors	20.23 ± 1.66	19.91 ± 1.65	0.91	.37
	Ability factors	13.05 ± 0.92	12.64 ± 1.07	1.89	.06
	Treatment factor	15.51 ± 1.28	16.00 ± 1.78	1.48	.14
SS		48.88 ± 2.42	48.49 ± 2.42	0.77	.45

Abbreviations: Cs, control group; GCBT, group cognitive behavioural therapy; HRSD, Hamilton Rating Scale for Depression; MMAS, Morisky Medication Adherence Scale™; SS, Stigma Scale.

a Is the *Z*‐value.

### 3.3 Comparison of scores and ranks between GCBTs and Cs after GCBT

Compared with Cs, GCBTs appeared to have lower scores in HRSD‐24, SS, and factor scores for SS, and higher scores in MMAS‐8™ after 8 weeks' GCBT. The differences between the two groups were statistically significant (*P* < .01). After 8 weeks' GCBT, the GCBTs' ranks in terms of treatment compliance were significantly better than those of Cs (*P* < .01). These results are shown in Table 4.

**Table 4:** Comparison of scores and ranks between GCBTs and Cs after GCBT(*M* ± *S*/*n* [%])

		GCBTs (*n* = 43)	Cs (*n* = 45)	*t*(Z)‐value	*P*‐value*
HRSD		11.12 ± 3.58	13.07 ± 2.54	2.96	.00
MMAS‐8		7.76 ± 0.39	5.73 ± 1.20	10.68	.00
Ranks	Good compliance	28 (65.12)	0 (0.00)		
	Moderate compliance	15 (34.88)	25 (55.56)	7.01^a^	.00
	Poor compliance	0 (0.00)	20 (44.44)		
SS	Social factors	16.53 ± 2.02	18.71 ± 2.31	4.70	.00
	Ability factors	9.79 ± 2.28	11.62 ± 1.28	4.61	.00
	Treatment factor	6.79 ± 1.37	13.56 ± 2.05	18.26	.00
SS		33.12 ± 3.25	43.89 ± 2.92	16.37	.00

Abbreviations: Cs, control group; GCBT, group cognitive behavioural therapy; HRSD, Hamilton Rating Scale for Depression; MMAS, Morisky Medication Adherence Scale™; SS, Stigma Scale.

a Is the *Z*‐value.

* *P*<.01.

### 3.4 Comparison of ranks in terms of therapeutic effects between GCBTs and Cs

After 8 weeks of GCBT, the GCBTs' ranks in terms of therapeutic effects were significantly better than those of Cs (*P* < .05). These results are shown in Table 5.

**Table 5:** Comparison of GCBTs' and Cs’ ranks in terms of therapeutic effects (*n*[%])

	Healing (RD ≥ 75%)	More progressive (75%>RD ≥ 50%)	Less progressive (50% > RD ≥ 25%)	Invalid (RD < 25%)
GCBTs (*n* = 43)	8 (18.60)	30 (69.77)	5 (11.63)	0 (0.00)
Cs (*n* = 45)	2 (4.44)	32 (71.11)	9 (20.00)	2 (4.44)
Z‐value	2.36			
*P*‐value	.02*			

Abbreviations: Cs, control group; GCBT, group cognitive behavioural therapy; RD, rate of deduction.

* *P* < 0.05.

## 4 DISCUSSION

Stigmatizing beliefs, such as that MDD is caused by insufficient self‐control or lack of willpower, often result in a sense of shame and embarrassment for MDD (Corrigan, 2004; Midgley et al., 2017). Research shows that patients with MDD can benefit from psychological interventions (De Jonge, Bockting, Kikkert, Bosmans, & Dekker, 2015; Driessen et al., 2013). Both stigma and poor treatment compliance can directly affect the therapeutic outcome of patients, and can even lead to negative emotional experiences such as feelings of frustration and uselessness, maladaptive coping behaviours such as concealment and avoidance and aggravated depressive symptoms (Martinez, Xu, & Hebl, 2018).

The purpose of this study was to explore the effects of GCBT in reducing stigma and improving treatment compliance for first‐episode MDD outpatients. The results of the study showed that there were no significant differences, in terms of demographic data and baseline level, between the GCBTs and Cs before the treatment; this indicated that the two groups had good homogeneity and comparability. After 8 weeks of GCBT, the GCBTs showed significant differences compared to the Cs in terms of HRSD‐24, MMAS‐8™, SS, SS factor score, treatment compliance and ranks of MMAS‐8™. The results indicated that GCBT can reduce stigma and improve the treatment compliance of patients with MDD and that this can ultimately lead to changes in the therapeutic effects on MDD.

The differences between GCBTs and Cs may have arisen because the GCBTs were in a group with patients suffering from the same disorder, and this resulted in a sense of belonging, acceptance, empathy, and concern; this, in turn, helped to eliminate feelings of inferiority, reduced stigma, and even benefited these participants through interpersonal interactions, experience‐sharing, altruism, and other factors. The experiences of the GCBTs helped them understand more about MDD, thus enabling them to develop coping strategies for their MDD symptoms; this reduced their feelings of stigma and increased their MDD treatment compliance (Huntley, Araya, & Salisbury, 2012). The results of this research validated the hypothesis that there are mutual effects among stigma, treatment compliance, and MDD symptoms (Figure 1).

Previous studies had found that GCBT had a significant effect on the treatment of MDD (Archer et al., 2016; Eiraldi et al., 2016; Strauss et al., 2018). Similarly, in this study, compared with the Cs, the GCBTs showed significant reductions in their HRSD scores, thus indicating that GCBT was effective for reducing depressive symptoms. Comparing the differences between GCBTs and Cs, statistical significances were found in terms of therapeutic effects by evaluating the RD of HRSD‐24 (ie, after the GCBT), and most of the participants were found to have acquired healing and progress. The treatment effects for the GCBTs were better than those of the Cs. While stigma and treatment compliance are closely related to the therapeutic outcomes of MDD treatments, this study's results may be related to the selected theme for the GCBT setting—reduce stigma and improve treatment compliance in participants with MDD. The pathogenesis of MDD is still unclear, and the therapeutic effects of existing treatment strategies have not been satisfactory. Although stigma is an obstacle in the path of recovery for most MDD patients, it has often been neglected by research, and MDD patients usually do not insist on treatment; instead, they often choose to avoid and even refuse treatment. Incorrect attitudes towards MDD and the poor treatment compliance behaviours of MDD patients have resulted in a low cure rate and easy recurrence. This situation is often the result of negative perceptions they hold about the disease and themselves. The stigma often comes from their feelings of frustration, failure of the ineffective response strategies, and a sense of guilt reflected in thoughts such as “I need to be responsible for the disease myself” (Carrara & Ventura, 2018; Latalova et al., 2014; Li, Li, & Ma, 2018; Tucker et al., 2013). However, receiving appropriate medical attention and psychotherapy can benefit MDD patients (Leuchs, Zinserling, & Schlosser‐Weber, 2014; Mohamed et al., 2017).

This study's results support the conclusion that GCBT can reduce stigma and improve MDD patients' treatment compliance, thus leading to a better partnership in the care process and greater improvements for treatment outcomes. These results can provide new directions for the psychological treatment of MDD and research regarding the relationships and underlying mechanisms between stigma, treatment compliance and MDD symptoms. Indeed, the current clinical research on GCBT is in its early stages. In the future, more topics regarding GCBT on MDD can be designed, and the clinical efficacy of GCBT can be measured by combining physiological, immune and functional effects to expand the clinical application of GCBT. Since GCBT can benefit more patients, it should be used more widely.

Although we conclude that GCBT can reduce stigma and improve treatment compliance in MDD patients, the present study has several limitations. The first was sampling bias: all participants were recruited from a single clinic. The second was lack of multiple comparison groups: there were no samples of patients receiving pharmacological treatment with add‐on psychotherapy other than GCBT or patients receiving placebo. The third was lack of results of repeated measurements and results of follow‐up measurements. Despite these limitations, this study is important, the results suggest that stigma and treatment compliance may be the cause of the treatment effect of patients with first‐episode MDD, which provide a new entry point for psychotherapy.

## Footnotes

*
The MMAS‐8 Scale, content, name, and trademarks are protected by US copyright and trademark laws. Permission for use of the scale and its coding is required. A license agreement is available from: MMAR, LLC. Donald E. Morisky, ScD, ScM, MSPH, 294 Lindura Ct.,Las Vegas, NV USA; dmorisky@gmail.com.

## References

[eip12841-bib-0001] Abiri, S. , Oakley, L. D. , Hitchcock, M. E. , & Hall, A. (2016). Stigma related avoidance in people living with severe mental illness (SMI): Findings of an integrative review. Community Mental Health Journal, 52, 251–261. 26668008 10.1007/s10597-015-9957-2

[eip12841-bib-0002] Archer, K. R. , Devin, C. J. , Vanston, S. W. , Koyama, T. , Phillips, S. E. , Mathis, S. L. , … Wegener, S. T. (2016). Cognitive‐behavioral‐based physical therapy for patients with chronic pain undergoing lumbar spine surgery: A randomized controlled trial. The Journal of Pain, 7, 76–89. 10.1016/j.jpain.2015.09.013 PMC4709178 26476267

[eip12841-bib-0003] Bahar, G. , Savas, H. A. , Nal, A. , Savas, E. , Hilal, K. , & Bahar, A. (2014). Reliability and validity of the Morisky medication adherence scale for bipolar mood disorder. Anadolu Psikiyatri Der, 15, 141–149.

[eip12841-bib-0004] Beck, J. S. (2011). Cognitive behavior therapy: Basics and beyond (2nd ed.). New York, NY: Guilford Press.

[eip12841-bib-0005] Butler, R. M. , Boden, M. T. , Olino, T. M. , Morrison, A. S. , Goldin, P. R. , Gross, J. J. , & Heimberg, R. G. (2018). Emotional clarity and attention to emotions in cognitive behavioral group therapy and mindfulness‐based stress reduction for social anxiety disorder. Journal of Anxiety Disorders, 55, 31–38. 29558650 10.1016/j.janxdis.2018.03.003 PMC5879018

[eip12841-bib-0006] Carrara, B. S. , & Ventura, C. A. A. (2018). Self‐stigma, mentally ill persons and health services: An integrative review of literature. Archives of Psychiatric Nursing, 32(2), 317–324. 29579531 10.1016/j.apnu.2017.11.001

[eip12841-bib-0007] Corrigan, P. (2004). How stigma interferes with mental health care. The American Psychologist, 59, 614–625. 15491256 10.1037/0003-066X.59.7.614

[eip12841-bib-0008] Corrigan, P. W. , Watson, A. C. , & Miller, F. E. (2006). Blame, shame, and contamination: The impact of mental illness and drug dependence stigma on family members. Journal of Family Psychology, 20, 239–246. 16756399 10.1037/0893-3200.20.2.239

[eip12841-bib-0009] De Jonge, M. , Bockting, C. L. , Kikkert, M. J. , Bosmans, J. E. , & Dekker, J. J. (2015). Preventive cognitive therapy versus treatment as usual in preventing recurrence of depression: Protocol of a multi‐centered randomized controlled trial. BMC Psychiatry, 15, 139. 26129694 10.1186/s12888-015-0508-8 PMC4487965

[eip12841-bib-0010] Driessen, E. , Van, H. L. , Don, F. J. , Peen, J. , Kool, S. , Westra, D. , … Dekker, J. J. (2013). The efficacy of cognitive‐behavioral therapy and psychodynamic therapy in the outpatient treatment of major depression: A randomized clinical trial. American Journal of Psychiatry, 170, 1041–1050. 24030613 10.1176/appi.ajp.2013.12070899

[eip12841-bib-0011] Eiraldi, R. , Power, T. J. , Schwartz, B. S. , Keiffer, J. N. , McCurdy, B. L. , Mathen, M. , & Jawad, A. F. (2016). Examining effectiveness of group cognitive‐behavioral therapy for externalizing and internalizing disorders in urban schools. Behavior Modification, 40(4), 611–639. 26872957 10.1177/0145445516631093 PMC5129163

[eip12841-bib-0012] Firmin, R. L. , Lysaker, P. H. , Luther, L. , Yanos, P. T. , Leonhardt, B. , Breier, A. , & Vohs, J. L. (2018). Internalized stigma in adults with early phase versus prolonged psychosis. Early Intervention in Psychiatry, 2, 1–7. 10.1111/eip.12553 29602244

[eip12841-bib-0013] Goffman, E. (1963). Stigma: Notes on the management of spoiled identity (pp. 5). Englewood Cliffs, NJ: Prentice Hall.

[eip12841-bib-0014] Hamilton, M. (1967). Development of a psychiatric rating scale for primary depression. British Journal of Clinical Psychology, 6, 278–296. 10.1111/j.2044-8260.1967.tb00530.x 6080235

[eip12841-bib-0015] Huntley, A. L. , Araya, R. , & Salisbury, C. (2012). Group psychological therapies for depression in the community: Systematic review and meta‐analysis. British Journal of Psychiatry, 200, 184–190. 22383765 10.1192/bjp.bp.111.092049

[eip12841-bib-0016] Kular, A. , Perry, B. I. , Brown, L. , Gajwani, R. , Jasini, R. , Islam, Z. , … Singh, S. P. (2018). Stigma and access to care in first‐episode psychosis. Early Intervention in Psychiatry, 10, 1–6. 10.1111/eip.12756 30411522

[eip12841-bib-0017] Latalova, K. , Kamaradova, D. , & Prasko, J. (2014). Perspectives on perceived stigma and self‐stigma in adult male participants with depression. Neuropsychiatric Disease and Treatment, 10, 1399–1405. 25114531 10.2147/NDT.S54081 PMC4122562

[eip12841-bib-0018] Leuchs, A. K. , Zinserling, J. , & Schlosser‐Weber, G. (2014). Estimation of the treatment effect in the presence of non‐compliance and missing data. Statistics in Medicine, 33(2), 193–208. 23873693 10.1002/sim.5924

[eip12841-bib-0019] Li, G. , Li, D. , & Ma, Y. (2018). The disease psychological characteristics and interventions of self‐focused attention. Medicine and Philosophy, 39, 67–80.

[eip12841-bib-0020] Martinez, L. R. , Xu, S. , & Hebl, M. (2018). Utilizing education and perspective taking to remediate the stigma of taking antidepressants. Community Mental Health Journal, 54, 450–459. 29022148 10.1007/s10597-017-0174-z

[eip12841-bib-0021] Midgley, N. , Parkinson, S. , Holmes, J. , Stapley, E. , Eatough, V. , & Target, M. (2017). “Id I bring it on myself?” An exploratory study of the beliefs that adolescents referred to mental health services have about the causes of their depression. European Child & Adolescent Psychiatry, 26, 25–34. 27207089 10.1007/s00787-016-0868-8 PMC5233729

[eip12841-bib-0022] Mohamed, S. , Johnson, G. R. , Chen, P. , Hicks, P. B. , Davis, L. L. , Yoon, J. , … Little, J. T. (2017). Effect of antidepressant switching vs augmentation on remission among patients with major depressive disorder unresponsive to antidepressant treatment the VAST‐D Randomized Clinical Trial. JAMA, 318(2), 132–145. 28697253 10.1001/jama.2017.8036 PMC5817471

[eip12841-bib-0023] Morisky, D. E. , Ang, A. , Krousel‐Wood, M. , & Ward, H. J. (2008). Predictive validity of a medication adherence measure in an outpatient setting. Journal of Clinical Hypertension, 10, 348–354. 18453793 10.1111/j.1751-7176.2008.07572.x PMC2562622

[eip12841-bib-0024] Okumura, Y. , & Ichikura, K. (2014). Efficacy and acceptability of group cognitive behavioral therapy for depression: A systematic review and meta‐analysis. Journal of Affective Disorders, 164, 155–164. 24856569 10.1016/j.jad.2014.04.023

[eip12841-bib-0025] Picco, L. , Lau, Y. , Pang, S. , Abdin, E. , Vaingankar, J. A. , Chong, S. A. , & Subramaniam, M. (2017). Mediating effects of self‐stigma on the relationship between perceived stigma and psychosocial outcomes among psychiatric outpatients: Findings from a cross‐sectional survey in Singapore. BMJ Open, 7, e01822. 10.1136/bmjopen-2017-018228 PMC5724097 28851803

[eip12841-bib-0026] Strauss, A. Y. , Huppert, J. D. , Simpson, H. B. , & Foa, E. B. (2018). What matters more? Common or specific factors in cognitive behavioral therapy for OCD: Therapeutic alliance and expectations as predictors of treatment outcome. Behaviour Research and Therapy, 105, 43–51. 29621650 10.1016/j.brat.2018.03.007 PMC5939572

[eip12841-bib-0027] Tang, F. , & Wen, Y. (2015). The relationship between psychological illness stigma and psychological help attitude. China Journal of Health Psychology, 23, 1495–1499.

[eip12841-bib-0028] Townsend, L. , Musci, R. , Stuart, E. , Ruble, A. , Beaudry, M. B. , Schweizer, B. , … Sawrtz, K. (2017). The association of school climate, depression literacy, and mental health stigma among high school students. The Journal of School Health, 87, 567–574. 28691174 10.1111/josh.12527 PMC5520658

[eip12841-bib-0029] Tucker, J. R. , Hammer, J. H. , Vogel, D. L. , Bitman, R. L. , Wade, N. G. , & Maier, E. J. (2013). Disentangling self‐stigma: Are mental illness and help‐seeking self‐stigmas different? The Counseling Psychologist, 60(4), 520–531. 10.1037/a0033555 23815629

[eip12841-bib-0030] Wang, Y. , Yuan, C. , & Sun, X. (2015). Group cognitive behavioral therapy for patients with mild depressive disorder and factors of therapeutic effect. Journal of Shanghai Jiaotong University(Medical Science), 35, 1136–1140.

[eip12841-bib-0031] Yang, A. , Wang, B. , Zhu, G. , Jiao, Z. , Fang, Y. , Tang, F. , … Zhong, M. (2014). Validation of Chinese version of the Morisky Medication Adherence Scale in participants with epilepsy. Seizure, 23(4), 295–299. 24484672 10.1016/j.seizure.2014.01.003

[eip12841-bib-0032] Zeng, Q. , He, Y. , & Tian, H. (2009). Development of scale of stigma in people with mental illness. Chinese Mental Health Journal, 23, 634–642.

[eip12841-bib-0033] Zhang, Y. (1998). Handbook of psychiatric assessment scale (2nd ed., pp. 35–39). Changsha, China: Hunan Science and Technology Press.

